# Elevation of NTN4 Expression and Its Possible Regulation by Tumor Necrosis Factor-Alpha (TNF-α) in a Rat Model of Rotator Cuff Tear

**DOI:** 10.7759/cureus.86324

**Published:** 2025-06-18

**Authors:** Kosuke Inoue, Kentaro Uchida, Ryo Tazawa, Mitsuyoshi Matsumoto, Tomonori Kenmoku, Yui Uekusa, Masashi Takaso

**Affiliations:** 1 Department of Orthopaedic Surgery, Kitasato University School of Medicine, Sagamihara, JPN

**Keywords:** mmp-3, netrin-4, rat model, rotator cuff tears, tnf-α

## Abstract

Introduction: Rotator cuff tears are a major cause of shoulder dysfunction and pain, particularly in older adults, with re-tear rates remaining high despite surgical advances. Persistent inflammation and dysregulated extracellular matrix (ECM) remodeling contribute to impaired healing. Tumor necrosis factor-α (TNF-α) is known to drive tendon inflammation, but the downstream mediators linking TNF-α signaling to matrix degradation remain incompletely understood. Netrin-4 (NTN4), a laminin-related protein, has been implicated in inflammatory and ECM-modulating processes. We hypothesized that NTN4 is upregulated following tendon tear in a TNF-α-dependent manner and contributes to sustained ECM degradation.

Methods: A rat infraspinatus and supraspinatus full-thickness tendon tear model was established, and tendon tissues were harvested at 0 (intact), 7, 14, 28, and 56 days post-injury (n = 8/time point). Gene expressions of *Ntn4*, *Il6*, *Tnfa*, *Mmp1*, *Mmp3*, and *Cxcl1* were quantified by qRT-PCR (quantitative real-time reverse transcription polymerase chain reaction). Primary rat tenocytes were stimulated in vitro with recombinant TNF-α (0-10 ng/mL) or NTN4 (0-500 ng/mL), and target gene expression and protein secretion were assessed by qRT-PCR and ELISA (enzyme-linked immunosorbent assay). Statistical comparisons were performed using Kruskal-Wallis with Dunn's post-hoc tests.

Results: In vivo, *Ntn4* expression significantly increased from day 14 onward (P < 0.001), peaking at day 28 and remaining elevated at day 56. *Tnfa* and *Il6* were upregulated earlier (days 7-28), and *Mmp3* showed sustained induction (days 7-28), while *Mmp1* rose later (day 28). In vitro, TNF-α induced *Ntn4* in a dose-dependent manner (P < 0.001). Exogenous NTN4 induced MMP-3 expression at both transcript and protein levels, while no significant changes were observed in MMP-1, TNF-α, or IL-6 protein levels under these experimental conditions.

Conclusions: Our findings identify NTN4 as a TNF-α-induced effector that contributes to sustained MMP-3-mediated matrix degradation following rotator cuff tear. By linking inflammatory cytokine signaling to prolonged ECM remodeling, the TNF-α/NTN4/MMP-3 axis may represent a potential therapeutic target for improving tendon healing and reducing re-tear risk.

## Introduction

Rotator cuff tears are among the most prevalent musculoskeletal injuries, affecting up to 30-50% of individuals over 60 years of age and representing a leading cause of shoulder pain and functional impairment [1,2]. Despite refinements in surgical techniques for massive tears, tendon re-tear rates remain high, often exceeding 40%, and incomplete healing has been attributed in part to persistent inflammation, aberrant neovascularization, and dysregulated extracellular matrix (ECM) remodeling [3-6].

Proinflammatory cytokines, particularly tumor necrosis factor-α (TNF-α), have been implicated in both clinical and experimental rotator cuff pathology. Serum TNF-α levels are elevated in patients with rotator cuff tears and correlate with sleep disturbance, suggesting that systemic inflammatory burden contributes to symptom severity [7]. Nagura et al. (2019), using a rat rotator cuff tear model, demonstrated that TNF-α regulates nerve growth factor (NGF) expression following rotator cuff tear [8]. In a similar rat model, Xiang et al. (2024) showed that local TNF-α blockade reduced pain-related behaviors and attenuated NGF upregulation, indicating that TNF-α drives nociceptive sensitization via NGF signaling [9]. Translational studies have also explored other tendon-derived mediators: apelin expression is upregulated in torn human rotator cuff samples and appears to modulate tenocyte metabolism and vascular responses, offering a potential therapeutic target [10]. However, attempts to predict tendon inflammatory gene expression, such as TNF-α, its receptor TNFR1, and downstream effectors like NSMAF and Casp3, based on clinical features have largely failed, underscoring the heterogeneous nature of tendon inflammation and the need for mechanistic biomarkers [11]. Collectively, these findings highlight a complex network in which TNF-α, NGF, apelin, and other mediators intersect to regulate inflammation, pain, and ECM turnover in rotator cuff injury.

Netrins are a family of secreted laminin-related proteins that modulate both neural and non-neural tissues through interactions with basement membrane components and cell-surface receptors [12-14]. Among them, Netrin‐4 (NTN4) binds laminin γ1 and various integrins to influence basement membrane assembly, angiogenesis, and cell-matrix adhesion [12,14]. In joint tissues, exogenous NTN4 stimulates infrapatellar fat pad- and synovium-derived fibroblasts to increase IL-6 and MMP production [15,16].

These findings suggest that NTN4 may contribute to the inflammatory microenvironment in joint diseases such as osteoarthritis. However, despite these insights, the role of NTN4 in tendon biology remains unexplored, and it remains unclear whether similar mechanisms operate in tendon tissues.

Given the established involvement of TNF-α in rotator cuff degeneration and NTN4's emerging role in joint inflammation and ECM remodeling, we hypothesized that NTN4 is a downstream mediator of TNF-α signaling in tendon tissue. Specifically, we posited that TNF-α induces NTN4 expression in tenocytes and that NTN4, in turn, promotes inflammatory and matrix-degrading responses contributing to tendon pathology.

To test this hypothesis, we employed both in vivo and in vitro approaches. In a rat model of full-thickness and infraspinatus tendons, we examined the temporal expression profile of NTN4 in tendon tissues to understand its role during the acute and subacute phases of injury. In parallel, we performed in vitro experiments using primary rat tenocytes stimulated with TNF-α to determine whether TNF-α directly regulates NTN4 expression and whether NTN4 modulates the expression of inflammatory and proteolytic factors such as IL-6 and MMP-3. This integrated approach allowed us to investigate the regulatory relationship between TNF-α and NTN4 and to assess the contribution of NTN4 to tendon matrix degradation.

## Materials and methods

Animals

Male Wistar rats (eight weeks old; Charles River Laboratories, Yokohama, Japan) were housed under a semi-barrier system at 23 ± 2 °C and 55 ± 10% humidity with a 12-hour light/dark cycle, with free access to commercial chow (CRF-1; Oriental Yeast Industry, Tokyo, Japan) and water. All procedures were conducted in accordance with the Science Council of Japan's guidelines and approved by the Kitasato University Animal Ethics Committee (approval no. 2024-116). A total of 48 rats were used in this study. Of these, 40 rats were used to establish the rotator cuff tear model and were sacrificed at five different time points (n = 8 per time point). In addition, tenocytes were isolated from four rats for in vitro NTN4 stimulation experiments and from another four rats for TNF-α stimulation experiments.

Rat rotator cuff tear model

Under anesthesia induced by intraperitoneal injection of Vetorphale, midazolam, and Domitor (1:1:3 ratio; 0.05 mL/100 g body weight), rats were placed in the lateral decubitus position. A 1-1.5 cm incision was made over the right shoulder, and the deltoid muscle was split longitudinally. A mini retractor (Fine Science Tools Inc., Vancouver, Canada) was used to gently retract the deltoid and expose the underlying rotator cuff tendons. Following a previously established model [8], the supraspinatus and infraspinatus tendons were sharply transected at their insertions on the greater tuberosity. The deltoid and skin were closed with 5-0 nylon sutures. The animals were returned to their cages with unrestricted activity. Approximately 2 mm segments of both supraspinatus and infraspinatus tendons were harvested at 0 (intact), 7, 14, 28, and 56 days post-tear (n = 8 per time point) for subsequent analyses.

RNA isolation and quantitative real-time PCR

The harvested tendon tissues were homogenized in TRIzol reagent (Invitrogen) using a Polytron PE1200 homogenizer (Kinematica AG, Malters, Switzerland) and centrifuged at 15,000 rpm for five minutes. The supernatant was combined with chloroform and processed through Qiagen Maxtract High Density tubes to isolate total RNA, while tenocyte cultures were processed using the Qiagen RNeasy Mini Kit (Hilden, Germany). RNA concentration and purity (A₂₆₀/A₂₈₀) were confirmed on a Denovix DS-11 spectrophotometer (DeNovix Inc., Wilmington, DE, USA), accepting only samples with ratios between 1.8 and 2.0. For cDNA synthesis, 500 ng of tendon RNA or 200 ng of tenocyte RNA was primed with random hexamer primers (65 °C, five minutes) and reverse-transcribed at 50 °C for 60 minutes using SuperScript III (Invitrogen) in First-Strand Buffer supplemented with DTT and RNase inhibitor; the reaction was terminated at 70 °C for five minutes.

Quantitative PCR was performed on a Bio-Rad CFX96 using 25 µL reactions containing 2 µL of cDNA, 200 nM of each gene-specific primer (Table 1), and 12.5 µL SYBR Premix Ex Taq™ (Takara; Takara Bio USA, Inc., San Jose, CA, USA). Cycling conditions were 95 °C for three minutes, followed by 40 cycles of 95 °C for 10 seconds and 60 °C for 30 seconds. Melt-curve analysis verified primer specificity. Relative transcript levels were calculated using the 2⁻ΔΔCt method with GAPDH as the internal control.

**Table 1:** Primer sequences used in this study bp: base pair

Gene		Sequence	bp
Ntn4	sense	TCGACATCAACGTGTGGGAG	198
	antisense	AGCTGAAAGGAAGGATCGCC	
Cxcl1	sense	GCCACACTCAAGAATGGTCG	91
	antisense	TGGGGACACCCTTTAGCATC	
Tnfa	Sense	CTCTTCTCATTCCCGCTCGT	104
	antisense	GGGAGCCCATTTGGGAACTT	
Il6	Sense	CCAGTTGCCTTCTTGGGACT	224
	antisense	TCTGACAGTGCATCATCGCT	
Mmp1	sense	CTCACACATTCCCACCAGGC	82
	antisense	TTGTCACTGTTGTCGGTCCA	
Mmp3	sense	TTGGCACAAAGGTGGATGCT	103
	antisense	TGGGTCACTTTCCCTGCATT	
Gapdh	sense	GACTCCAAGAACTGCACCGA	172
	antisense	TCCGGTACACGATCACTCCT	

Isolation and culture of rotator cuff-derived tenocytes

Additional eight-week-old Wistar rats were euthanized, and rotator cuff tendons were minced and digested in 0.1% collagenase I (Wako Pure Chemical Industries, Osaka, Japan) at 37 °C for one hour. The resulting cell suspension was filtered, centrifuged, and plated in α-MEM (minimum essential medium) (Gibco™, Thermo Fisher Scientific, Waltham, MA, USA) supplemented with 10% fetal bovine serum (Gibco). Tenocytes were maintained at 37 °C in a humidified 5% CO₂ incubator and used at passage 1.

TNF-α and NTN4 treatment

After serum starvation in α-MEM containing 1% FBS for 12 hours, tenocytes were exposed either to recombinant rat NTN4 (R&D Systems, Minneapolis, MN, USA) at final concentrations of 0, 50, or 500 ng/mL or to recombinant rat TNF-α (PeproTech, Cranbury, NJ, USA) at 0, 1, or 10 ng/mL. Treatments were applied for three, six, or 24 hours, after which cells were harvested for RNA extraction and qRT-PCR as described above, using the primers listed in Table 1.

Statistical analysis

All group comparisons were performed using the Kruskal-Wallis test followed by Dunn's multiple-comparison test. A P-value < 0.05 was considered statistically significant. All statistical analyses were performed using IBM SPSS Statistics for Windows, Version 28 (Released 2021; IBM Corp., Armonk, New York).

## Results

Gene expression of NTN4, inflammatory cytokines, MMPs, and CXCL1 following rotator cuff injury

**Figure 1:**
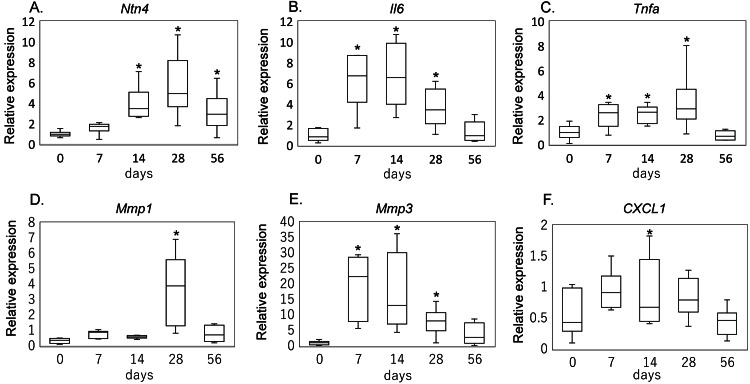
Temporal gene expression changes following rotator cuff injury Box-and-whisker plots depict mRNA expression levels of (A) Ntn4, (B) Il6, (C) Tnfa, (D) Mmp1, (E) Mmp3, and (F) Cxcl1 in tendon tissue at 0 days (intact control) and at 7, 14, 28, and 56 days post-injury. Statistical significance (*) is indicated for comparisons versus day 0 (P < 0.05). Statistical analysis was performed using the Kruskal–Wallis test with Dunn's post-hoc test.

Compared with intact tendons (day 0), Ntn4 expression showed no significant change at day 7 (P = 0.257) but rose markedly thereafter, with highly significant increases at days 14 and 28 (both P < 0.001) and remained elevated at day 56 (P = 0.007) (Figure 1A). Similarly, IL-6 mRNA was robustly upregulated early, peaking at day 7 (P < 0.001) and day 14 (P < 0.001), and was still significantly higher at day 28 (P < 0.017) before returning to baseline levels by day 56 (P = 0.797) (Figure 1B). TNF-α followed a comparable but somewhat attenuated pattern, with significant induction at day 7 (P = 0.012), day 14 (P = 0.008), and day 28 (P = 0.002), then subsiding to non-significance by day 56 (P = 0.564) (Figure 1C). Of the matrix metalloproteinases, MMP-1 did not reach significance until day 28 (P < 0.001) and showed only trends at earlier (day 7, P = 0.066; day 14, P = 0.152) and later (day 56, P = 0.129) time points (Figure 1D). In contrast, MMP-3 was strongly induced as early as day 7 (P < 0.001) and remained elevated through day 14 (P < 0.001) and day 28 (P < 0.013) before declining toward baseline by day 56 (P = 0.257) (Figure 1E). Finally, CXCL1 was modestly increased at day 7 (P = 0.043) but thereafter returned to baseline at days 14, 28, and 56, with no further significant changes (Figure 1F).

Effects of exogenous NTN4 on tenocytes

**Figure 2:**
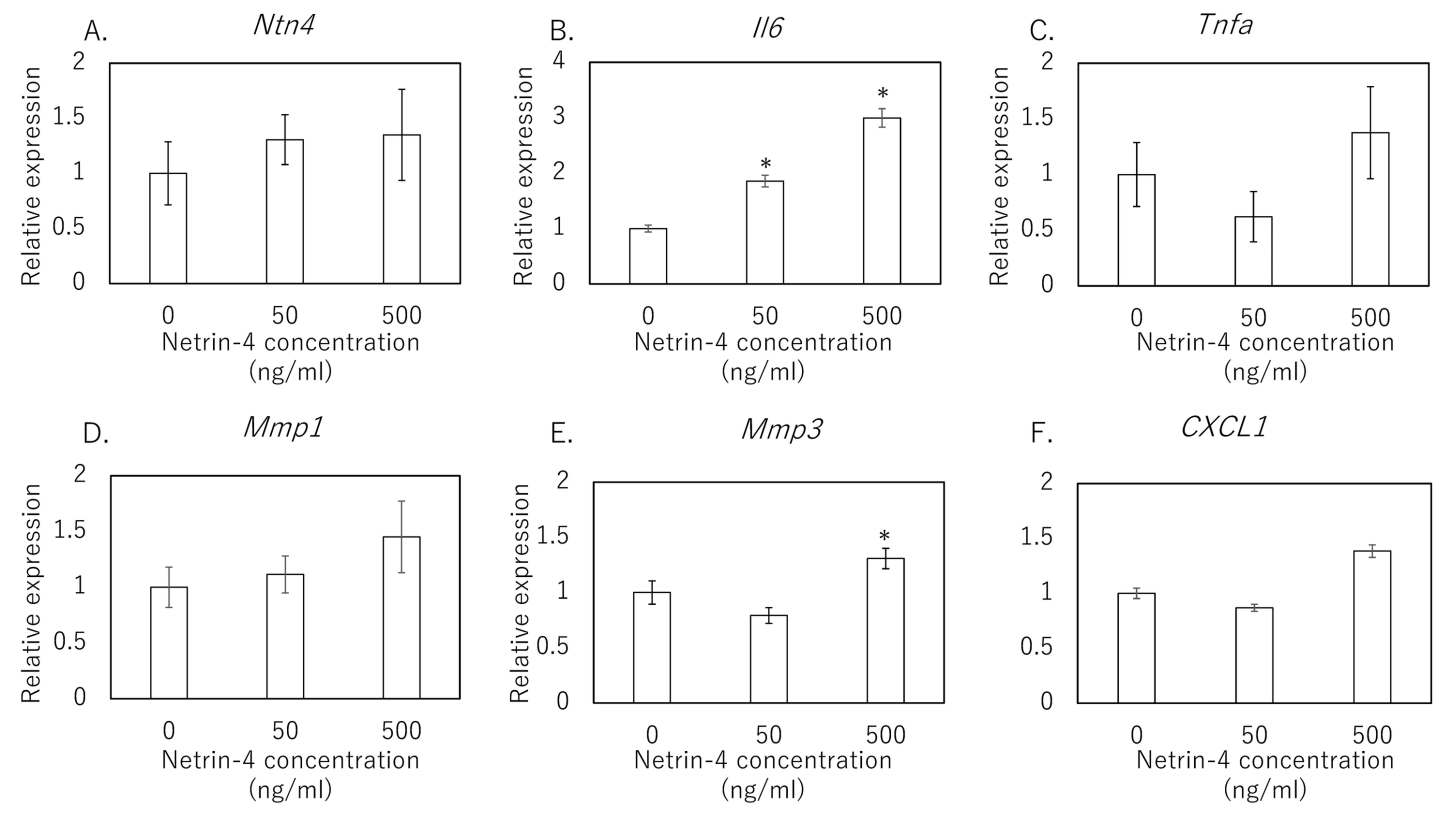
Effect of NTN4 on gene expression of Ntn4, inflammatory cytokines, MMPs, and CXCL1 in rat rotator cuff-derived tenocytes mRNA expression levels of (A) Ntn4, (B) Il6, (C) Tnfa, (D) Mmp1, (E) Mmp3, and (F) Cxcl1 were measured in primary tenocytes isolated from rat rotator cuff tendons following stimulation with recombinant NTN4. Data are presented as mean ± SEM. Statistical significance (*) is indicated for comparisons versus control (0 ng/mL) (P < 0.05). Statistical analysis was performed using the Kruskal–Wallis test with Dunn's post-hoc test. SEM: standard error of the mean

To assess whether NTN4 directly regulates tenocyte inflammatory and matrix-remodeling responses, cells were treated for 24 hours with vehicle (0 ng/mL), 50 ng/mL, or 500 ng/mL recombinant human NTN4. At the mRNA level, Ntn4 itself showed a non-significant trend toward elevation at 50 ng/mL (P = 0.054) and 500 ng/mL (P = 0.112) (Figure 2A). IL-6 transcripts were robustly induced at both 50 ng/mL (P < 0.002) and 500 ng/mL (P < 0.001) (Figure 2B), whereas TNF-α remained unchanged (50 ng/mL, P = 0.803; 500 ng/mL, P = 0.726) (Figure 2C). MMP-1 expression did not differ from vehicle at either dose (50 ng/mL, P = 0.937; 500 ng/mL, P = 0.397) (Figure 2D). In contrast, MMP-3 mRNA increased significantly at 500 ng/mL (P < 0.001) with a non-significant trend at 50 ng/mL (P = 0.172) (Figure 2E), and CXCL1 was unaffected (50 ng/mL, P = 0.289; 500 ng/mL, P = 0.092) (Figure 2F).

**Figure 3:**
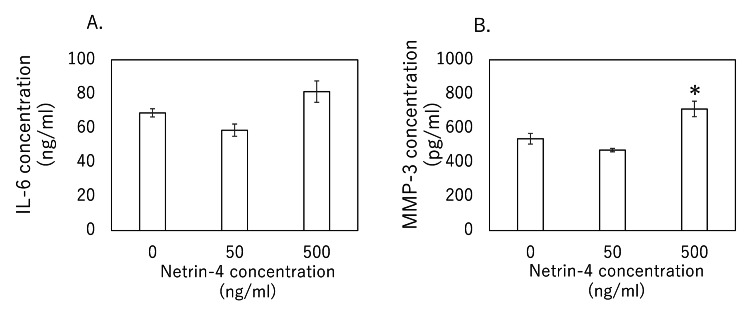
Protein levels of IL-6 and MMP-3 in NTN4-stimulated rat rotator cuff-derived tenocytes Protein concentrations of (A) IL-6 and (B) MMP-3 were measured by ELISA in culture supernatants of rat rotator cuff-derived tenocytes following stimulation with recombinant NTN4. Data are presented as mean ± SEM. Statistical significance (*) is indicated for comparisons versus control (0 ng/mL) (P < 0.05). Statistical analysis was performed using the Kruskal–Wallis test with Dunn's post-hoc test. ELISA: enzyme-linked immunosorbent assay; SEM: standard error of the mean

The corresponding ELISA (enzyme-linked immunosorbent assay) of conditioned supernatants revealed no significant change in IL-6 protein at either 50 ng/mL (P = 0.282) or 500 ng/mL (P = 0.168) (Figure 3A), whereas MMP-3 secretion rose in parallel with its transcript only at 500 ng/mL (P = 0.011; 50 ng/mL, P = 0.355) (Figure 3B). These findings indicate that NTN4-driven transcriptional upregulation of MMP-3 is coupled to increased protease release, whereas IL-6 mRNA induction does not translate into elevated extracellular cytokine under these conditions.

Dose-dependent induction of NTN4 by TNF-α in tenocytes

**Figure 4:**
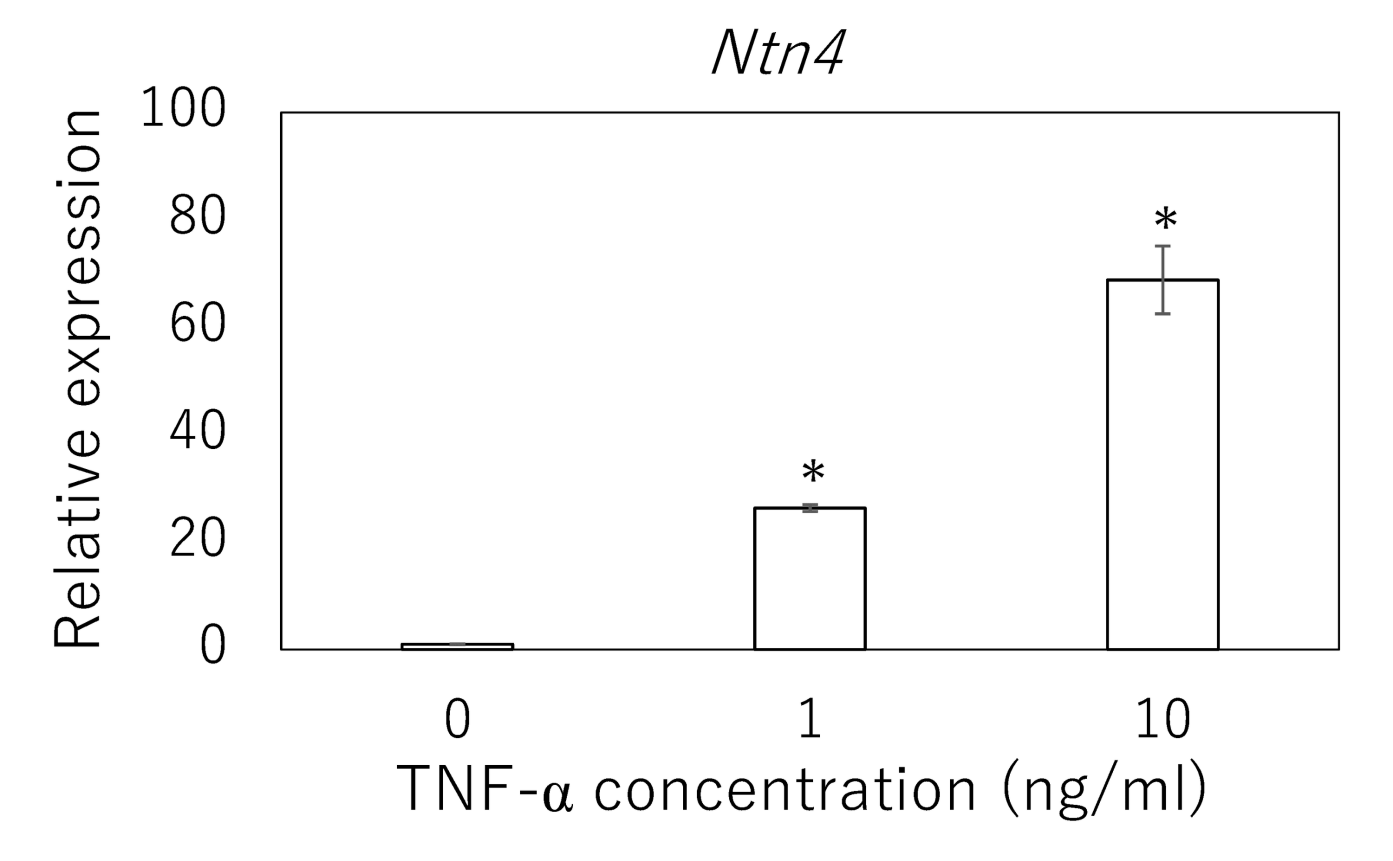
Effect of TNF-α stimulation on Ntn4 expression in rat rotator cuff-derived tenocytes mRNA expression levels of Ntn4 were measured in rat rotator cuff-derived tenocytes following stimulation with TNF-α. Data are presented as mean ± SEM. Statistical significance (*) is indicated for comparisons versus control (0 ng/mL) (P < 0.05). Statistical analysis was performed using the Kruskal–Wallis test with Dunn's post-hoc test. SEM: standard error of the mean; TNF-α: tumor necrosis factor-alpha

Cultured tenocytes exposed to TNF-α for 24 hours exhibited a robust, dose-dependent increase in Ntn4 mRNA levels. Relative to vehicle-treated controls (1.00 ± 0.12), treatment with 1 ng/mL TNF-α drove Ntn4 expression to 26.32 ± 1.32 (mean ± SD), while 10 ng/mL further amplified expression to 68.82 ± 12.63 (Figure 4). The statistical analysis revealed a significant overall effect of TNF-α concentration on Ntn4 induction (P < 0.001), and post-hoc comparisons confirmed that both 1 ng/mL and 10 ng/mL treatments significantly increased Ntn4 levels compared to the vehicle (P < 0.001 for each).

## Discussion

In the rat supraspinatus tear model, IL-6 and TNF-α transcripts were rapidly upregulated by day 7 and remained elevated through day 28, whereas Ntn4 mRNA did not rise until day 14 and stayed high through day 56. MMP-3 expression increased as early as day 7 and was sustained through day 28, while MMP-1 induction emerged later at day 28. In vitro, TNF-α stimulation of tenocytes produced a dose-dependent surge in Ntn4, and exogenous NTN4 in turn selectively upregulated MMP-3 transcription and secretion without altering TNF-α or IL-6 protein levels. Together, these findings indicate that, following rotator cuff tear, an early TNF-α-driven increase in NTN4 may serve as a key intermediary that prolongs MMP-3-mediated matrix degradation, thereby linking acute inflammation to sustained tendon remodeling.

Our in vitro experiments provide mechanistic insight into this process, demonstrating that TNF-α stimulation induces a robust, dose- and time-dependent increase in NTN4 expression in tenocytes. This positions NTN4 as a downstream effector of TNF-α signaling in tendon cells. Such cytokine-neurotrophin interactions have been previously implicated in musculoskeletal pathologies. For instance, in tendon and joint tissues, TNF-α is known to promote the expression of neurotrophic factors that contribute to nociceptive sensitization [9,15]. Our findings extend this concept to tendon ECM regulation, suggesting that TNF-α-driven NTN4 expression may serve dual roles in modulating both pain pathways and matrix remodeling.

Importantly, we demonstrated that recombinant NTN4 is sufficient to upregulate MMP-3 expression at both transcript and protein levels in tenocytes, whereas MMP-1 levels remained unchanged, indicating a selective effect on MMP-3. MMP-3, a broad-spectrum matrix metalloproteinase, plays a pivotal role in ECM degradation by cleaving proteoglycans, collagens, and fibronectin and by activating other MMPs. Promoter polymorphisms in MMP-3 have been associated with increased rotator cuff tear susceptibility, underscoring a genetic contribution to MMP-mediated tendon integrity [17]. In a rotator cuff repair model, pharmacologic inhibition of MMPs improved tendon-to-bone healing, suggesting that early MMP suppression may enhance structural outcomes [18]. By establishing a direct link between NTN4 and MMP-3 expression, our study provides a plausible mechanism whereby inflammatory signals may sustain pathological ECM degradation. Given that NTN4 has also been implicated in regulating angiogenesis and inflammation in other disease contexts [15,16], it may serve as an integrative node connecting inflammation, vascular remodeling, and matrix turnover in tendon pathology.

Several limitations of our study should be considered. First, we did not perform histological or biomechanical analyses of the injured tendons. Consequently, we cannot directly correlate the molecular findings with structural tissue remodeling, scar formation, or mechanical function. Future studies incorporating such assessments are warranted to clarify whether modulation of the TNF-α/NTN4/MMP axis influences long-term healing outcomes. Second, while our focus was on MMP-3, we did not comprehensively assess the broader protease-inhibitor balance, including other MMPs and tissue inhibitors of metalloproteinases (TIMPs). Elucidating this network will be important for understanding the overall dynamics of ECM homeostasis in tendon healing. Third, the intracellular signaling pathways downstream of NTN4 receptor engagement remain to be characterized. Pathways such as PI3K/Akt, MAPK, and NF-κB are plausible candidates, and dissecting these mechanisms could identify actionable molecular targets. Finally, we did not evaluate the effects of NTN4 on non-tenocyte cell types, which may also contribute to tendon inflammation and remodeling.

In summary, our findings identify NTN4 as a TNF-α-induced mediator that contributes to sustained MMP-3-driven matrix degradation in the context of rotator cuff injury. By bridging inflammatory cytokine signaling and proteolytic ECM remodeling, NTN4 emerges as a potential therapeutic target for mitigating pathological tendon degeneration. Targeting this pathway may offer novel avenues to improve tendon healing and reduce the incidence of re-tears, which remain a significant clinical challenge.

While our model provides insight into the temporal dynamics and mechanistic role of NTN4 in an acute rotator cuff tear setting, it is important to recognize that most clinical cases involve chronic degenerative tendon injuries rather than acute trauma. The development of reliable chronic tendon injury models in animals remains technically challenging due to difficulties in replicating the prolonged inflammatory and degenerative changes seen in human pathology. We acknowledge this as a limitation of our study. To date, there are no published reports demonstrating elevated NTN4 expression in human tendon disease samples. Therefore, although our findings highlight a potentially important molecular mechanism in tendon matrix degradation, further validation in clinical tissue samples is necessary to determine the relevance of this pathway in human rotator cuff pathology. Future studies using human tendon specimens will be required to assess whether the TNF-α/NTN4/MMP-3 axis identified in this model is conserved in chronic tendon disease and whether NTN4 could serve as a biomarker or therapeutic target in the clinical setting.

## Conclusions

In conclusion, our study reveals that, following rotator cuff tear, an early TNF-α-driven rise in NTN4 sustains MMP-3-mediated extracellular matrix degradation well beyond the acute inflammatory phase. In vitro validation confirms that TNF-α directly induces NTN4 in tenocytes and that NTN4 upregulates MMP-3 transcription and secretion. These data position NTN4 as a critical intermediary linking inflammation to pathological matrix remodeling. By targeting the TNF-α/NTN4/MMP-3 axis, future therapeutic strategies may attenuate early proteolysis, promote healthier tendon repair, and ultimately reduce re-tear rates in rotator cuff injuries.

## References

[1] Tempelhof S, Rupp S, Seil R (1999). Age-related prevalence of rotator cuff tears in asymptomatic shoulders. J Shoulder Elbow Surg.

[2] Yamazaki H, Ochiai N, Kenmoku T (2014). Assessment of pain-related behavior and pro-inflammatory cytokine levels in the rat rotator cuff tear model. J Orthop Res.

[3] Galatz LM, Ball CM, Teefey SA, Middleton WD, Yamaguchi K (2004). The outcome and repair integrity of completely arthroscopically repaired large and massive rotator cuff tears. J Bone Joint Surg Am.

[4] Millar NL, Wei AQ, Molloy TJ, Bonar F, Murrell GA (2009). Cytokines and apoptosis in supraspinatus tendinopathy. J Bone Joint Surg Br.

[5] Voloshin I, Gelinas J, Maloney MD, O'Keefe RJ, Bigliani LU, Blaine TA (2005). Proinflammatory cytokines and metalloproteases are expressed in the subacromial bursa in patients with rotator cuff disease. Arthroscopy.

[6] Zumstein MA, Jost B, Hempel J, Hodler J, Gerber C (2008). The clinical and structural long-term results of open repair of massive tears of the rotator cuff. J Bone Joint Surg Am.

[7] Cho CH, Kim DH, Baek EH, Kim DH (2021). Serum levels of TNF-α are increased in patients with rotator cuff tear and sleep disturbance. Diagnostics (Basel).

[8] Nagura N, Kenmoku T, Uchida K, Nakawaki M, Inoue G, Takaso M (2019). Nerve growth factor continuously elevates in a rat rotator cuff tear model. J Shoulder Elbow Surg.

[9] Xiang L, Deng H, Zhou S (2024). Effects of TNF-α on behaviour and inflammation in rats with rotator cuff injury through NGF. Discov Med.

[10] Nakawaki M, Kenmoku T, Uchida K, Arendt-Nielsen L, Nagura N, Takaso M (2023). Expression of apelin in rotator cuff tears and examination of its regulatory mechanism: a translational study. Cureus.

[11] Struzik S, Czarkowska-Paczek B, Wyczalkowska-Tomasik A, Maldyk P, Paczek L (2021). Selected clinical features fail to predict inflammatory gene expressions for TNF-α, TNFR1, NSMAF, Casp3 and IL-8 in tendons of patients with rotator cuff tendinopathy. Arch Immunol Ther Exp (Warsz).

[12] Larrieu-Lahargue F, Welm AL, Thomas KR, Li DY (2011). Netrin-4 activates endothelial integrin α6β1. Circ Res.

[13] Reuten R, Patel TR, McDougall M (2016). Structural decoding of netrin-4 reveals a regulatory function towards mature basement membranes. Nat Commun.

[14] Schneiders FI, Maertens B, Böse K (2007). Binding of netrin-4 to laminin short arms regulates basement membrane assembly. J Biol Chem.

[15] Tsukada A, Uekusa Y, Ohta E (2025). Association between synovial NTN4 expression and pain scores, and its effects on fibroblasts and sensory neurons in end-stage knee osteoarthritis. Cells.

[16] Uekusa Y, Mukai M, Tsukada A (2024). Elevated netrin-4 expression and its action in infrapatellar fat pad. Int J Mol Sci.

[17] Assunção JH, Godoy-Santos AL, Dos Santos MC, Malavolta EA, Gracitelli ME, Ferreira Neto AA (2017). Matrix metalloproteases 1 and 3 promoter gene polymorphism is associated with rotator cuff tear. Clin Orthop Relat Res.

[18] Bedi A, Kovacevic D, Hettrich C, Gulotta LV, Ehteshami JR, Warren RF, Rodeo SA (2010). The effect of matrix metalloproteinase inhibition on tendon-to-bone healing in a rotator cuff repair model. J Shoulder Elbow Surg.

